# Cyclin D1 and p16 expression in recurrent nasopharyngeal carcinoma

**DOI:** 10.1186/1477-7819-4-62

**Published:** 2006-09-05

**Authors:** Ho-Sheng Lin, Gerald J Berry, Zijie Sun, Willard E Fee

**Affiliations:** 1 Department of Surgery, John D. Dingell VA Medical Center, 4646 John R. Street, Detroit, MI 48201, USA; 2 Department of Otolaryngology, Wayne State University, 4201 St. Antoine, 5 E University Health Center, Detroit, MI 48201, USA; 3 Department of Pathology, Stanford University Medical Center, Stanford, CA 94305, USA; 4 Department of Urology, Stanford University Medical Center, Stanford, CA 94305, USA; 5 Department of Otolaryngology, Stanford University Medical Center, Stanford, CA 94305, USA

## Abstract

**Background:**

Cyclin D1 and p16 are involved in the regulation of G1 checkpoint and may play an important role in the tumorigenesis of nasopharyngeal carcinoma (NPC). Previous studies have examined the level of expression of cyclin D1 and p16 in primary untreated NPC but no such information is available for recurrent NPC. We set out in this study to examine the expression level of cyclin D1 and p16 in recurrent NPC that have failed previous treatment with radiation +/- chemotherapy.

**Patients and methods:**

A total of 42 patients underwent salvage nasopharyngectomy from 1984 to 2001 for recurrent NPC after treatment failure with radiation +/- chemotherapy. Twenty-seven pathologic specimens were available for immunohistochemical study using antibodies against cyclin D1 and p16.

**Results:**

Positive expression of cyclin D1 was observed in 7 of 27 recurrent NPC specimens (26%) while positive p16 expression was seen in only 1 of 27 recurrent NPC (4%).

**Conclusion:**

While the level of expression of cyclin D1 in recurrent NPC was similar to that of previously untreated head and neck cancer, the level of p16 expression in recurrent NPC samples was much lower than that reported for previously untreated cancer. The finding that almost all (96%) of the recurrent NPC lack expression of p16 suggested that loss of p16 may confer a survival advantage by making cancer cells more resistant to conventional treatment with radiation +/- chemotherapy. Further research is warranted to investigate the clinical use of p16 both as a prognostic marker and as a potential therapeutic target.

## Background

Nasopharyngeal carcinoma (NPC) is a prevalent malignancy in Southeast Asia with reported incidence rate ranging from 10 to 53 cases per 100,000 persons per year [1]. Although it is a relatively uncommon cancer in western countries with an incidence rate of less than 1 case per 100,000, it poses a significant health problem in regions of the US where there is large population of Asians. External beam radiation therapy is considered the primary mode of treatment for previously untreated NPC. This is mainly due to the high degree of sensitivity of this tumor to radiation as well as the anatomic constraints for surgical access in this highly complex region. Despite advances in diagnostic and treatment modalities, loco-regional failure is still significant with reported rates of 15.6% to 58% [1,2]. The addition of chemotherapy concurrent with radiation has been shown to improve survival in patients with NPC [3].

Although the molecular events responsible for the pathogenesis of NPC remain to be elucidated, the final common pathway appears to be a disruption of the mechanisms involved in the regulation of cell cycle progression, leading to uncontrolled cell proliferation. The most critical point in cell cycle regulation is the G_1 _checkpoint. It is here that complex interactions take place to determine whether the cell will exit the cell cycle and go into a quiescent state (G_0_) or enter into the S phase and proceed with cell division [4]. These complex interactions involve a large number of regulatory proteins such as cyclins, cyclin-dependent kinases (CDKs), and CDK-inhibitors. Among the cyclins involved in G1 phase, cyclin D1 appears to be most strongly implicated in human carcinogenesis. Cyclin D1, through its interaction with cyclin-dependent kinase-4 or 6 (CDK-4/6), forms a complex that inactivates the tumor suppressor protein retinoblastoma (pRb) through phosphorylation [4]. Phosphorylation of pRb releases transcription factors such as E2Fs which then activate a series of events that allow entry into S-phase and cell division [4]. An increased level of cyclin D1 expression has been reported in a number of malignancies [5] including esophageal, ovarian, breast, uterine, colon, lung, prostate, lymphoma, as well as head and neck cancers. Between 30% to 83% of head and neck squamous cell carcinoma (HNSCC) were reported to be associated with cyclin D1 over-expression [6,7]. The progression of cells from G1 to S phase, on the other hand, is blocked by a potent tumor suppressor protein, p16, which acts to disrupt the cyclin D1/CDK-4/6 complex. Deletions, mutations, or methylation of the p16 gene has been implicated in the development of a variety of human malignancies, including head and neck cancer [6,8].

The disruption of the "Retinoblastoma/cyclin D1/p16 pathway" involved in the regulation of the G1 checkpoint also appears to play an important role in the tumorigenesis of NPC. Over-expression of cyclin D1 and/or loss of p16 has been reported in several studies on NPC. These studies found an increased level of cyclin D1 expression in 30% [7] to 66% [9] and the lack of expression of p16 in 40% to 70% of previously untreated NPC specimens [9-12].

Although a large body of data exists on cyclin D1 and p16 expressions in HNSCC including NPC, to the best our knowledge, no previous studies have been reported on the expression level of these proteins in recurrent HNSCC. We set out in this study to examine the expression level of cyclin D1 and p16 in recurrent NPC that have failed previous treatment with radiation +/- chemotherapy.

## Methods

### Patients

A total of 42 patients underwent salvage nasopharyngectomy by one of the investigators (WEF) at Stanford University Medical Center from 1984 to 2001 for recurrent NPC after treatment failure with radiation +/- chemotherapy. The clinical, demographic, and results of salvage nasopharyngectomy on the first 37 patients have been published previously [13]. Between 2000 and 2001, 5 additional patients underwent nasopharyngectomy, bringing the total number of patients to 42. A search through the archival tissue bank at Stanford Medical Center identified 27 pathologic specimens which were available for immunohistochemical study using antibodies against cyclin D1 and p16. The other 15 specimens were unavailable for this study due to exhaustion of the limited amount of pathological specimen during the original pathologic diagnosis.

The surgical approach for the salvage nasopharyngectomy depended on the location of the tumor but included intraoral, transpalatal, and sublabial transmaxillary approaches. Isolation of the internal carotid artery and cranial nerves IX, X, and XII to the skull base was performed. Tumors were removed en bloc when possible. Any recurrent neck disease was addressed with a neck dissection. There was no evidence of distant metastasis in any of these study patients. At the time of salvage surgery, 20 patients have previously been treated with 1 course of radiation, 2 patients with 2 courses of radiation, 2 patients with 2 courses of radiation plus chemotherapy, and 3 patients with a single course of radiation plus chemotherapy.

### Tissue specimens

**Figure 1:**
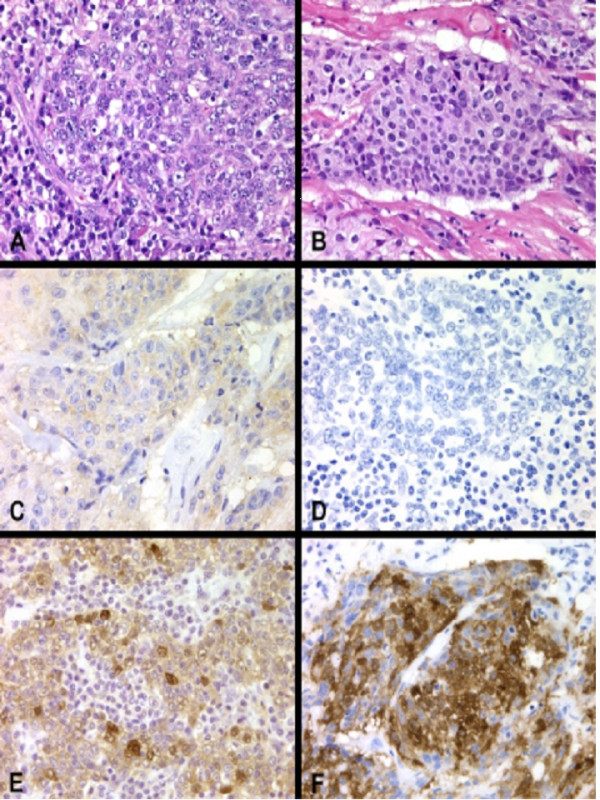
Panel A: Lymphoepithelioma type of nasopharyngeal carcinoma (H&E × 400); Panel B: Poorly differentiated nasopharyngeal carcinoma (H&E × 400); Panel C: Grade 0 staining for Cyclin D1 (× 400); Panel D: Grade 0 staining for p16; Panel E: Grade 3+ staining for Cyclin D1 (× 400); Panel F: Grade 3+ staining for p16 (× 400)

The formalin-fixed, paraffin-embedded surgical specimens removed from these 27 patients were used for this study. Tissue blocks from the surgical margin were selected in order to include both the normal and cancer tissues in the same slide. The blocks were sectioned at 5 μm and mounted on poly-L-Lysine coated slides. The sections were stained with H&E and reviewed by a single pathologist (GJB) to confirm the diagnosis of NPC. Most of the specimens were classified as undifferentiated (WHO III) and poorly differentiated NPC (WHO II) (Figure 1, Panel A and B).

### Immunohistochemistry for cyclin D1 and p16

Using the Biogenex i1000, deparaffinization and microwave antigen retrieval were performed on the tissue sections. This was followed by immunostaining using the Dako Autostainer. Briefly, the slides were incubated in 0.3% hydrogen peroxide and blocked using a non-serum based blocking agent. The slides were then incubated with primary monoclonal mouse anti-cyclin D1 antibody (dilution 1:25; Zymed, South San Francisco, CA) and monoclonal anti-p16 antibody (F-12) (dilution 1:40; Santa Cruz Biotechnology, Santa Cruz, CA) for 60 minutes at room temperature. Following the primary incubation, the slides were incubated with biotinylated goat anti-mouse antibody (Santa Cruz Biotechnology, Santa Cruz, CA) for 10 minutes followed by incubation with strepavidin-peroxidase reagent for 10 minutes. Finally, the slides were visualized using DAB and counterstained with Mayer's hematoxylin.

### Analysis of level of expression

Immunostaining of cyclin D1 and p16 was evaluated by a board-certified pathologist (GJB). Ten high power fields were successively examined using standard light microscopy. Cytoplasmic reactivity was disregarded as nonspecific and only staining of tumor nuclei was scored as positive. A specimen was scored as negative or no staining (0) when < 5% of the tumor cells exhibited nuclear staining. A specimen was considered positive when > 5% of the tumor cells exhibited nuclear staining. The 5% cut-off for positivity was chosen in order to be consistent with other similar studies on p16 and cyclin D immunostaining in the literature. Non-uniform staining of p16 and cyclin D1 was expected since the expression of these 2 proteins in a particular tumor cell is dependent on the phase of cell cycle with a peak expression in G1 phase. The intensity of the positive immunostains were then graded semi-quantitatively as 1+ (positive/weak staining), 2+ (positive/moderate staining), and 3+ (positive/strong staining) (additional file 1).

### Statistical analysis

Overall disease-free survival was calculated using the Kaplan-Meier program from SPSS 14.0 for Windows (SPSS, Chicago, IL). Significance in survival outcome between different groups of patients in this study population was analyzed by the Log-rank test (Mantel-Cox).

## Results

Twenty-seven recurrent NPC specimens were examined in this study. The average age at the time of salvage nasopharyngectomy was 51 years (range, 27 to 72 years). There were 18 men and 9 women. Thirteen patients were Asian and 14 were non-Asians. Patients were restaged using the 1998 AJC Cancer Staging Manual and 16 were staged as recurrent (r)T1, 2 as rT2, 5 as rT3, and 4 as rT4. Other relevant clinicopathological characteristics of these patients were shown in additional file 1.

The results of the immunohistochemical staining of these 27 recurrent NPC specimens were presented on additional file 1. Cyclin D1 over-expression was limited to tumor cells and no immunostaining was noted on the surrounding normal epithelium of the nasopharynx. Thus, the normal epithelium served as internal negative control. On the other hand, normal tissue stained positive for p16 and was used as internal positive control. Representative samples of cyclin D1 and p16 immunostaining were shown in Figure 1. Absence of staining for cyclin D1 and p16 were illustrated in panel C and D respectively. In Panel E and F, 3+ immunostaining was demonstrated for cyclin D1 and p16 respectively. Of the 27 recurrent NPC specimens, 7 (26%) stained positive for cyclin D1 proteins while only 1 (4%) demonstrated immunostaining for p16. Of the 7 specimens that stained positive for cyclin D1 protein, 1 stained strongly (3+), 3 stained moderately (2+), and 3 stained weakly (1+).

**Figure 2:**
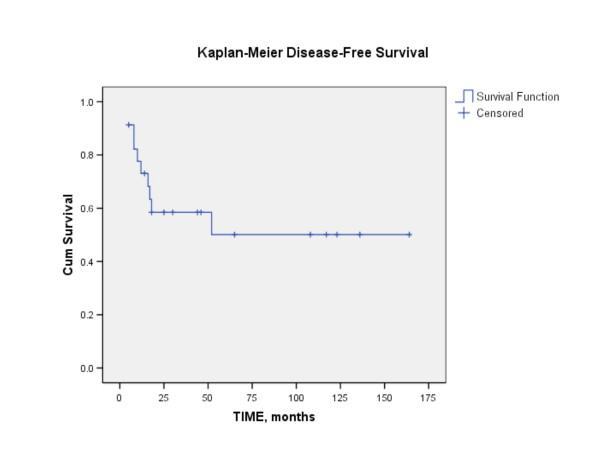
Kaplan-Meier analysis showing the disease-free survival following salvage nasopharyngectomy. The 5-year disease free survival was 50%.

**Figure 3:**
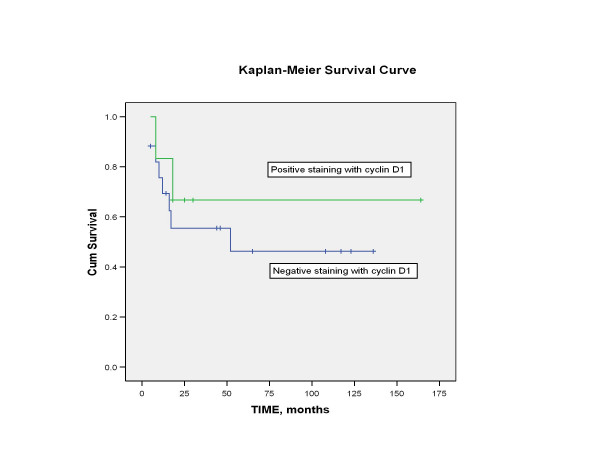
Kaplan-Meier analysis showing the disease-free survival of patients whose tumor specimens stained positive for cyclin D1 and patients whose tumor specimens stained negative. Based on Log-rank test, there was no statistically significant difference in disease-free survival between these 2 groups based on cyclin D1 expression (p = 0.518).

**Figure 4:**
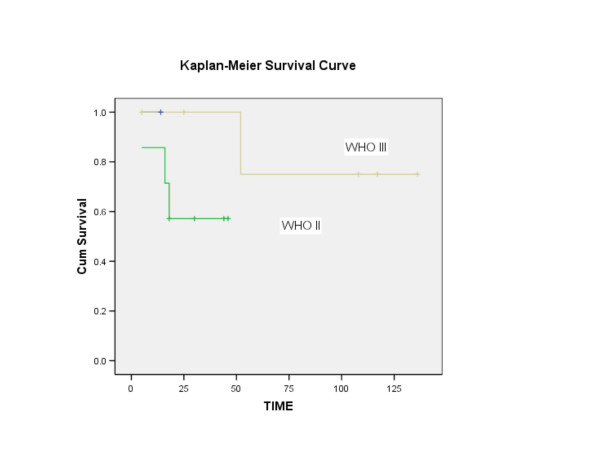
Kaplan-Meier analysis showing the disease-free survival of patients based on the WHO classification. There was no statistically significant difference in disease-free survival among these groups of patients (p = 0.243).

**Figure 5:**
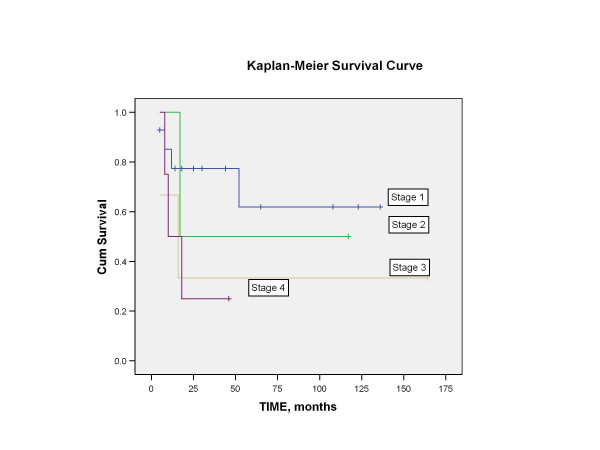
Kaplan-Meier analysis showing the disease-free survival of patients based on the staging at the time of salvage nasopharyngectomy. There was no statistically significant difference in disease-free survival among these 4 groups of patients (p = 0.347).

**Figure 6:**
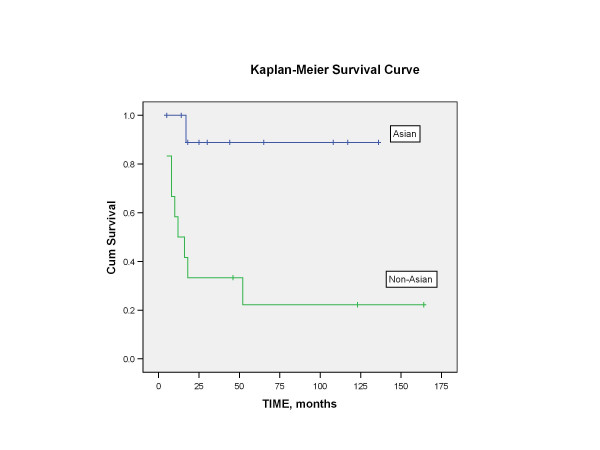
Kaplan-Meier analysis showing the disease-free survival of Asian (A) versus non-Asian (NA) patients. There was a statistically significant difference in the 5-year disease-free survival between Asian patients (88.9%) and non-Asian patients (22.2%) with p = 0.003 based on the Log-rank test.

Length of follow-up ranged from 5 months to 164 months, with mean of 45 months. Four patients were lost to follow-up and were not included in the survival analysis. At the time of last contact, patients were classified as no evidence of disease (NED), alive with disease (AWD), or died of disease (DOD). The 5-year disease-free survival following salvage nasopharyngectomy for the 23 patients (excluding the 4 patients that were lost to follow-up) was 50% (Figure 2). Using the Log-rank test, no statistical significance in disease-free survival was found to correlate with variables such as gender, staining with cyclin D1 (Figure 3), staining with p16, WHO classification (Figure 4), or recurrent stage (Figure 5). However, there was a statistically significant difference (p = 0.003) in 5-year disease-free survival between Asian (88.9%) and non-Asian patients (22.2%) (Figure 6).

## Discussion

In this study, we examined the expression level of cyclin D1 and p16 in recurrent NPC that have failed previous treatment with radiation +/- chemotherapy. These specimens were procured at the time of salvage nasopharyngectomy. We found over-expression of cyclin D1 in 26% and the lack of expression of p16 in 96% of recurrent NPC specimens. While the level of over-expression of cyclin D1 (26%) in recurrent NPC specimens was similar to the levels of over-expression (30%[7] to 66%[9]) found in primary untreated NPC, the lack of expression of p16 in recurrent NPC specimens (96%) was significantly higher than those typically reported for primary untreated NPC (40% to 70%) [9-12].

There are a number of studies that attempted to correlate the level of expression of cyclin D1 and p16 in cancer with prognosis. Several investigators reported association between over-expression of the proto-oncogene cyclin D1 with worse prognosis in terms of shorter survival and increased incidence of recurrence [14-16]. This poor prognosis in tumors over-expressing cyclin D1 was found to be independent of other clinicopathological parameters such as tumor stage, nodal or distant metastasis, or degree of differentiation [14-16]. Other studies however either failed to show this association between over-expression of cyclin D1 and poor clinical outcome [17] or found a reverse association between over-expression of cyclin D1 and poor prognosis. In a series of 65 patients, Hwang et al [9] reported that loss of cyclin D1 was closely related to local recurrence after radiation therapy for NPC. Our data also failed to support the association between over-expression of cyclin D1 with increased risk of recurrence. If over-expression of cyclin D1 was associated with increased risk of recurrence, we would expect to find a higher proportion of recurrent NPC specimens to over-express cyclin D1 compared to previously untreated NPC. However, the level of over-expression of cyclin D1 in recurrent NPC specimens found in this study (26%) was not higher, but slightly lower than those reported for previously untreated NPC (30% to 66%) [7,9,18].

The other interesting finding from this study was the lack of p16 expression in almost all (96%) of the recurrent NPC specimens. This was in contrast to the 40% to 70% reported rate of p16 loss in previously untreated NPC (table 1). This high proportion of recurrent cancer with loss of p16 activity suggested that p16 loss may confer survival advantage by making these cancer cells more resistant than their p16 positive counterparts to conventional treatment with radiation +/- chemotherapy. Other investigators have also reported on the association between loss of p16 expression and worse prognosis. Hwang et al [9] reported on a statistically significant correlation (p = 0.047) between absence of p16 expression with higher rate of local recurrence in NPC. In another study involving 84 NPC samples, Makitie et al [12] found a statistically significant association (p = 0.022) between absence of p16 expression and inferior overall survival rate. The absence of p16 expression found in nearly all of our recurrent NPC samples in this study seemed to corroborate the finding of these investigators. Taken together, these findings would suggest that the loss of p16 tumor suppressor proteins in NPC may confer resistance to radiotherapy and may be associated with higher rate of failure after therapy with radiation +/- chemotherapy.

**Table 1:** Four different studies showing that between 40% to 70% of previously untreated NPC specimens have loss of p16 expression.

**Investigators**	**Number of NPC specimens examined**	**Percent of NPC with loss of p16 staining**
**Hwang et al [13]**	65	65%
**Makitie et al [16]**	84	70%
**Huang et al [15]**	74	42%
**Baba et al [14]**	20	40%
**Lin et al [this study]**	27	96%

This is in contrast to the 96% of recurrent NPC found in this study to have loss of p16 expression.

Finally, it is interesting to note that Asian patients, following treatment with salvage nasopharyngectomy, have a statistically significant better 5-year disease-free survival compared to Caucasians and Hispanics (p = 0.003) (Figure 5). The reason for this difference is unclear. The Asian and non-Asian group did not differ significantly in their level of cyclin D1 expression. Four out of 13 Asians and 3 out of 14 non-Asians stained positive for cyclin D1.

Although this is the first study looking at the expression levels of cyclin D1 and p16 in recurrent NPC, this study has several limitations. First, pre-irradiated NPC specimen were not available for use as control due to the fact that most of these patients were referred from outside institutions (in many cases, from institutions outside of US) after treatment failure with radiation therapy. The unavailability of pre-irradiated NPC specimen raised the issue of whether the lack of expression of p16 in these recurrent NPC samples was pre-existing (pre-treatment) or the result of selection pressure from treatment. The data presented in this study will not be able to answer this question. Our assumption here was that these recurrent tumors would have the same staining characteristics showing absence of p16 expression even before treatment with radiation. Irrespective of whether the p16 expression was lost during tumor transformation (before treatment) or during treatment with radiation, this marked reduction of p16 in recurrent NPC specimen underscored the importance of p16 alteration in NPC and provided support for therapeutic strategies that aim to restore p16 expression. There have been pre-clinical studies which demonstrated the ability of exogenously introduced p16 to inhibit proliferation and induce apoptosis in NPC cell culture [19]. Another limitation of this study was the small number of cases (n = 27) examined. However, both the low prevalence of NPC in the US and the reluctance of most head and neck surgeons to surgically salvage recurrent NPC made access to a large number of surgically removed recurrent NPC specimen available for immunohistochemical study difficult.

## Conclusion

In this study, we have determined the level of expression of cyclin D1 and p16 in recurrent NPC that have failed primary treatment with radiation +/- chemotherapy. While the level of expression of cyclin D1 in recurrent NPC was similar to that of previously untreated HNSCC, almost all of the recurrent NPC samples lacked expression of p16 protein. This finding suggested that the loss of p16 tumor suppressor protein in NPC may be associated with higher rate of failure after treatment with radiation +/- chemotherapy. Thus, p16 expression level appeared to be a clinically useful prognostic marker in NPC and warranted further investigations. Further, therapeutic strategies targeting the p16 pathway may be a promising approach to improve the treatment outcome of NPC. Several promising studies have demonstrated the ability of exogenously introduced p16 to inhibit proliferation and induce apoptosis in NPC cell culture [19].

## Competing interests

The author(s) declare that they have no competing interests.

## Authors' contributions

**HSL **conceived the study, design, acquisition of data, analysis, and interpretation of data.

**GB **carried out, analyzed, and interpreted the histopathology and immunoassays.

**ZS **participated in the design, acquisition of data, analysis, and interpretation of data.

**WEF **conceived the study and participated in its design and coordination and revising it critically for important intellectual content.

All authors read and approved the final manuscript.

## Supplementary Material

Additional file 1 Expression levels of cyclin D1 and p16 in the 27 recurrent NPC specimens examined. A specimen was scored as negative or no staining (0) when < 5% of the tumor cells exhibited nuclear staining. A specimen is considered positive when > 5% of the tumor cells exhibited nuclear staining. The intensity of the positive immunostains was then graded semi-quantitatively as 1+ (positive/weak staining), 2+ (positive/moderate staining), and 3+ (positive/strong staining). Click here for file
