# Supplementary material for: Cyclin D1 and p16 expression in recurrent nasopharyngeal carcinoma
Source: World J Surg Oncol. 2006 Sep 5;4:62. doi: 10.1186/1477-7819-4-62 (PMC1569377; doi:10.1186/1477-7819-4-62)
Supplement: Additional file 1 — Expression levels of cyclin D1 and p16 in the 27 recurrent NPC specimens examined. A specimen was scored as negative or no staining (0) when < 5% of the tumor cells exhibited nuclear staining. A specimen is considered positive when > 5% of the tumor cells exhibited nuclear staining. The intensity of the positive immunostains was then graded semi-quantitatively as 1+ (positive/weak staining), 2+ (positive/moderate staining), and 3+ (positive/strong staining). [file 1477-7819-4-62-S1.doc]

| **#** | **Age** | **Race** | **Sex** | **TNM AJCC** | **WHO** | **rTNM AJCC 1998** | **Recur Stage** | **p16** | **cD1** | **Prior Tx** | **Salvage Surgery** | **Time to event** | **Last follow-up (Event)** |
| --- | --- | --- | --- | --- | --- | --- | --- | --- | --- | --- | --- | --- | --- |
| 1 | 53 | A | M | T1N0M0 | 1 | rT1N0M0 | 1 | 0 | 0 | XRT 7/95, Chemorad 1/01 | 10/01 | 14 | NED 12/02 |
| 2 | 64 | A | M | T3N0M0 | 2 | rT1N0M0 | 1 | 0 | **1+** | XRT 1995 | 6/01 | 18 | NED 12/02 |
| 3 | 49 | W | M | T2bN2bM0 | 2 | rT3N0M0 | 3 | **3+** | 0 | XRT 11/98, chemo/IMRT 6/00 | 2/01 | 16 | DOD 6/02 |
| 4 | 66 | W | M | T4N0M0 | 2 | rT4N0M0 | 4 | 0 | **2+** | XRT 10/98 | 12/00 | 18 | AWD 6/02 |
| 5 | 58 | A | M | T3N0M0 | 2 | rT3N0M0 | 3 | 0 | **1+** | Chemorad 1993, nasopharyngectomy 2/96 | 6/00 | 30 | NED 12/02 |
| 6 | 38 | A | F | T3N2bM0 | 2 | rT1N0M0 | 1 | 0 | 0 | XRT 12/96 | 3/99 | 44 | NED 11/02 |
| 7 | 55 | W | M | T3N0M0 | 2 | rT4N0M0 | 4 | 0 | 0 | Chemorad 8/97 | 11/98  stereo-XRT | 46 | NED 9/02 |
| 8 | 40 | A | M | T3N2M0 | 3 | rT1N0M0 | 1 | 0 | **2+** | Chemorad 7/95 | 12/96 | 25 | NED 1/99 |
| 9 | 34 | A | M | T1N0M0 | 2 | rT1N0M0 | 1 | 0 | 0 | XRT | 3/95 | NA | LFU |
| 10 | 67 | W | M | T1N0M0 | 2 | rT1N0M0 | 1 | 0 | 0 | XRT | 11/94 | NA | LFU |
| 11 | 44 | W | F | T2N2aM0 | 3 | rT1N0M0 | 1 | 0 | 0 | XRT 4/94 | 8/94 | 52 | AWD, lung mets,10/99 |
| 12 | 51 | A | M | T1N0M0 | 3 | rT1N0M0 | 1 | 0 | 0 | XRT 11/86,  2^nd^ XRT 1989 | 12/93 | 108 | NED 12/02 |
| 13 | 60 | A | M | NA | 2 | rT3N1M0 | 3 | 0 | **3+** | XRT | 11/93 | NA | LFU |
| 14 | 72 | W | M | T1N2cM20 | NA | rT1N0M0 | 1 | 0 | 0 | XRT | 11/93 | 12 | DOD |
| 15 | 38 | A | F | NA | 3 | rT1N1M0 | 2 | 0 | 0 | XRT | 3/93 | 117 | NED 12/02 |
| 16 | 61 | A | F | T1N0M0 | 3 | rT1N0M0 | 1 | 0 | 0 | XRT | 11/92 | NA | LFU |
| 17 | 70 | H | M | NA | NA | rT1N0M0 | 1 | 0 | 0 | XRT 1991 | 9/92 | 65 | NED@ death 2/98 |
| 18 | 62 | H | M | T2N0M0 | NA | rT1N0M0 | 1 | 0 | 0 | XRT 1987 | 8/92 | 123 | NED 11/02 |
| 19 | 28 | A | F | NA | 3 | rT1N0M0 | 1 | 0 | 0 | XRT 1988  2^nd^ XRT 1989 | 9/91 | 136 | NED 1/03 |
| 20 | 44 | H | M | T1N0M0 | NA | rT4N0M0 | 4 | 0 | 0 | XRT 1986 | 11/89 | 10 | DOD |
| 21 | 53 | W | M | T4N3bM0 | NA | rT4N0M0 | 4 | 0 | 0 | XRT 1987 | 11/89 | 8 | DOD |
| 22 | 47 | W | F | T2N0M0 | NA | rT3N0M0 | 3 | 0 | **1+** | XRT 1985  Surgery 7/88 | 5/89 | 164 | NED 1/03 |
| 23 | 66 | H | M | T1N0M0 | NA | rT1N0M0 | 1 | 0 | 0 | XRT 11/87 | 2/89 | 5 | DOD |
| 24 | 28 | H | M | T1N2M0 | NA | rT1N0M0 | 1 | 0 | **2+** | XRT 10/87 | 12/88 | 8 | DOD |
| 25 | 39 | A | M | T2N0M0 | 3 | rT1N0M0 | 1 | 0 | 0 | XRT 1986 | 11/87 | 5 | NED@ death 4/88 |
| 26 | 33 | A | M | T3N2bM0 | NA | rT2N1M0 | 2 | 0 | 0 | XRT 1/86 | 3/87 | 17 | DOD |
| 27 | 47 | W | F | T1N2aM0 | 2 | rT3N0M0 | 3 | 0 | 0 | XRT 2/86 | 6/87 | 5 | DOD |

**A = Asian, W = White, H = Hispanic, M = Male, F = Female, NED = no evidence of disease, DOD = died of disease, LFU = lost to follow-up, AWD = alive with disease, NA = Information not available**
